# Relationship between Cytomegalovirus Viremia and Long-Term Outcomes in Kidney Transplant Recipients with Different Donor Ages

**DOI:** 10.3390/microorganisms11020458

**Published:** 2023-02-11

**Authors:** Davide Diena, Anna Allesina, Fabrizio Fop, Alberto Mella, Rossana Cavallo, Cristina Costa, Caterina Dolla, Ester Gallo, Francesco Giuseppe De Rosa, Antonio Lavacca, Roberta Giraudi, Filippo Mariano, Luigi Biancone

**Affiliations:** 1 Renal Transplant Center “A. Vercellone”, Nephrology, Dialysis, and Renal Transplant Division, “Città Della Salute e Della Scienza” Hospital, Department of Medical Sciences, University of Turin, 10126 Turin, Italy; 2 Renal Unit, Santa Croce e Carle Hospital, 12100 Cuneo, Italy; 3 Microbiology and Virology Unit, University of Turin, 10126 Turin, Italy; 4 Department of Medical Sciences, Infectious Diseases, University of Turin, A.O.U. Città Della Salute e Della Scienza di Torino, 10126 Turin, Italy

**Keywords:** cytomegalovirus, renal transplantation, long-term outcomes, risk factors, donor age, cumulative incidence function

## Abstract

Objectives: To explore the Cytomegalovirus (CMV) burden on the long-term post-transplant course in different donor ages, we evaluated the incidence and risk factors for CMV in our kidney-transplanted patients (KTs) with extensive adoption of expanded-criteria donors (ECDs). Methods: Retrospective evaluation of 929 consecutive first KTs (49.5% receiving an organ from a donor ≥ 60 years) performed between 01-2003 and 12-2013. Overall survival was estimated using Kaplan–Meier curves; cumulative incidence function was additionally analyzed to consider the potential role of death with a functioning graft as a competitive event with graft dysfunction and to avoid overestimation. Apart from regular DNAemia monitoring in all patients, prophylaxis was adopted in high-risk groups (D+/R− or recipients of anti-thymocyte globulin induction), with pre-emptive therapy in the remaining groups. Results: CMV incidence was 19.5% (4–34.9% according to serostatus combination: D−/R−, D−/R+, D+/R+, D+/R−). Donor and recipient age, recipient pre-transplant hypertension, DR antigen compatibility, cold ischemia time, and post-transplant early complications, including rejection, urologic and renal artery stenosis, and lower renal function and proteinuria ≥ 0.5 g/day at one year after KT were associated with CMV. CMV determined lower death-censored graft survival (DCGS) (*p* < 0.01), with a prominent effect in R+ (*p* < 0.01) and without impact in R− (*p* = 0.32 in D−/R− and *p* = 0.006 in D+/R−). Interestingly, CMV occurrence influenced DCGS only in KTs who received grafts from donors < 50 or 50–69 years old (*p* < 0.01), while it was not significant with older donors (*p* = 0.07). The analysis of the cumulative incidence of graft loss accounting for death as a competing risk confirmed all these findings. In multivariate analysis, CMV replication/disease in the first year was an independent predictor for DCGS (HR 1.73 [1.3–2.3]). Conclusions: In a large population with extensive ECD adoption, CMV viremia in the first year demonstrates its harmful effect with an independent role for graft loss and significant impact among R+ recipients and KTs with donors < 70 years.

## 1. Introduction

Late failure of kidney transplants (KTs) represents a significant clinical problem that limits kidney transplantation duration. Several authors agree that late graft loss could be related to early, and not even immunologic, injuries (e.g., in the first 6–12 months after transplantation), which may lead to a maladaptive repair and subsequent alloimmune response, causing renal dysfunction, fibrosis, and at least organ failure [1]. Indeed, the rapid identification of at-risk groups may allow clinicians to improve long-term outcomes.

In this context, Cytomegalovirus (CMV), the most common viral infection after kidney transplantation, acquires a central role in its direct progression to CMV clinical syndrome and end-organ disease, but also for its indirect effects, including increased morbidity/mortality, acute and chronic allograft rejection, atherosclerosis, and diabetes [2,3,4].

Early CMV infection/disease incidence significantly differed based on donor-recipient serostatus: seronegative patients (R−) who received an organ from a seronegative donor (D−) had a lower risk. Seropositive recipients (R+) represent the vast majority (50–90%) worldwide and have an intermediate probability of CMV reactivation or superinfection; in (R−) KTs with CMV-IgG-positive donors (D+) primary disease and active replication with severe disease may often occur, and guidelines highly recommend a 3–6-month prophylaxis regimen with ganciclovir or valganciclovir [5]. Low-risk recipients (D−/R−) usually only underwent monitoring for clinical signs or symptoms [6]. Among other risk factors related to CMV replication/disease, studies mentioned donor and recipient age, renal disease, coinfection with other viruses like BK polyomavirus, the net rate of immunosuppression, and induction with anti-thymocyte globulins (ATGs). The correlation between CMV and biopsy-proven acute rejection is, to date, still a matter of debate [7].

Although several studies showed a significant correlation between CMV serostatus and disease with mortality and graft loss [8,9], the authors of a recent meta-analysis concluded that further studies are required to quantify the burden of CMV in adult KTs appropriately [7].

This study aims to evaluate CMV replication/disease incidence in our large cohort of KTs with a significant number of extended-criteria donors (ECDs) and assess its impact on long-term outcomes and the correlation of CMV with other known risk factors.

## 2. Materials and Methods

In this retrospective observational cohort study, we included all deceased donor grafts performed at the Turin University Renal Transplant Center “A. Vercellone” from January 2003 to December 2013; multi-organ grafts, dual kidney transplantation, and KTs who received previous transplants were excluded to limit confounding factors and homogenize the study population. To assess the difference between recipients of standard vs. marginal kidneys, we stratified the population according to donor ages (<50 years, between 50 and 69 years, and ≥70 years). Follow-up ended in November 2021. The local Ethical Committee approved this study (Comitato Etico Interaziendale A.O.U. Città Della Salute e Della Scienza di Torino-A.O. Ordine Mauriziano-A.S.L. Città di Torino, resolution 1449/2019 on 11/08/2019—“TGT observational study”).

All data were extracted from the recipients’ scheduled clinical visits and hospital admissions. D+/R− recipients and all KTs receiving ATG induction underwent CMV prophylaxis with valganciclovir for six and two months, respectively. The duration of prophylaxis could be modified in a small number of cases based on clinical judgment.

After transplantation, CMV viremia was regularly monitored in all recipients, irrespective of serostatus and donor/recipient CMV matching. The control schedule was biweekly in the first three months, monthly in the fourth month, and then every two months until one year after transplantation. Further controls were performed on a clinical basis.

CMV DNAemia was detected in whole blood using a commercially available real-time PCR assay (CMV-ELITe MGB^®^ kit, ELITechGroup, Milan, Italy). CMV replication is defined as DNAemia > 1160 UI/mL (=2000 copies/mL). This threshold was considered susceptible to pre-emptive therapy with oral valganciclovir to achieve the complete negativization of viremia. Intravenous ganciclovir use was limited to severe forms of CMV disease or in cases without response to valganciclovir.

Discrete data were described as percentages and analyzed with Pearson’s Χ^2^ or, for small samples, with Fisher’s exact test. The distribution of continuous variables was analyzed with the Kolmogorov–Smirnov test. Continuous variables were described as mean ± standard deviation when normal and as median with interquartile ranges when non-normally distributed. When appropriate, Mann–Whitney, Kruskal–Wallis, *t*-test, or variance analysis with a Bonferroni post hoc test were used to analyze the difference between groups. Kaplan–Meier (KM) curves analyzed cumulative graft and patient survival. A univariate model for the main clinically chosen covariates was adopted to identify significant predictors (level α = 0.05, log-rank test), followed by a multivariate analysis fitted with significant univariate variables.

To consider the potential role of death with a functioning graft as a competitive event with graft dysfunction and to avoid overestimation compared to the traditional Kaplan–Meier, we also calculated the cumulative incidence function [10]. Gray’s test assessed the statistical significance of the difference in the cumulative incidences of competing events among groups.

SPSS software was adopted for all the analyses (IBM Corp. Released 2021. IBM SPSS Statistics for Windows, Version 28.0.1.0, IBM Corp., Armonk, NY, USA). 

Competing risk analyses were conducted using R Statistical Software (v4.2.2; R Core Team 2022) and theR package cmprsk (v2.2-11). The significance level was α < 0.05.

## 3. Results

Nine hundred twenty-nine consecutive KTs with a mean follow-up of 10.51 years (5.38–11.43) were included in our analysis; among them, 460 (49.5%) received an organ from a donor ≥ 60 years. In this population, CMV replication/disease in the first year after transplant occurred in 181/929 (19.5%).

We observed that these patients had higher recipient and donor median age, higher rates of pre-transplant hypertension, lower DR antigen compatibility, and more prolonged cold ischemia than patients with no history of CMV viremia. Moreover, CMV was associated with the main early complications, including acute rejection, cardiovascular and urologic complications, infections, and renal artery stenosis (Table 1 and Table 2).

Regarding immunosuppressive therapy, patients with CMV replication/disease had a lower percentage of mTOR inhibitors as part of their maintenance therapy one year after transplantation (9.4% vs. 15.6%; *p* = 0.03).

**Figure 1:**
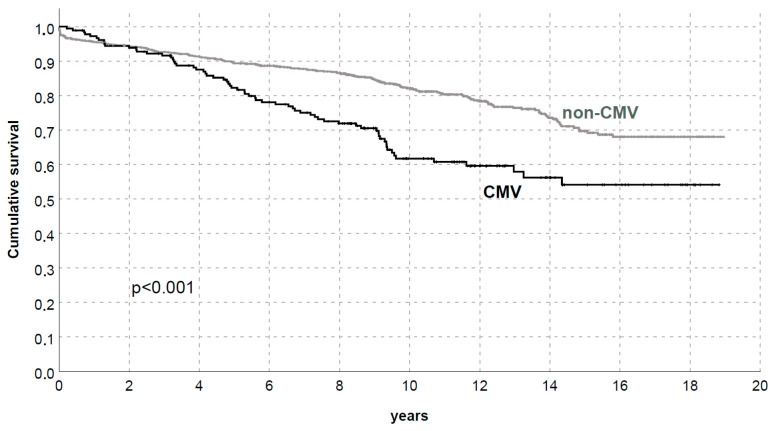
Death-censored graft survival by CMV replication/disease in the first year after transplantation.

**Figure 2:**
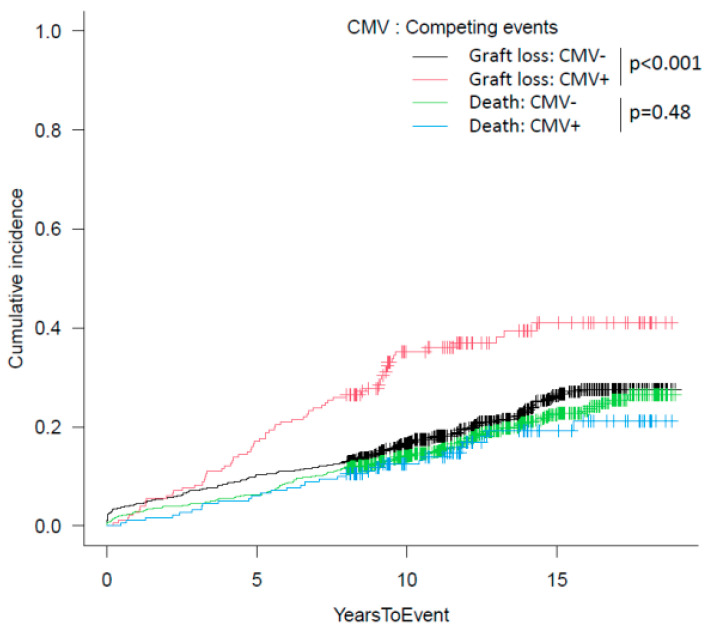
Cumulative incidence of graft loss, accounting for death as a competing risk, in patients with/without CMV replication/disease in the first year after transplantation.

We then investigated the CMV impact on patient/graft survival in our population: 5- and 10-year death-censored graft survival (DCGS) was 89.4/82.2% and 82.2/61.7% in KTs without/with CMV in the first year after transplant, respectively (*p* < 0.01 for all time-points, Figure 1). Cumulative incidence of graft loss accounting for death as a competing risk confirmed the negative impact of CMV (*p* < 0.001, Figure 2).

Patient survival did not differ between groups (90.8/91.7% at five years and 80.1/79.3% at ten years, *p* = 0.17).

**Figure 3:**
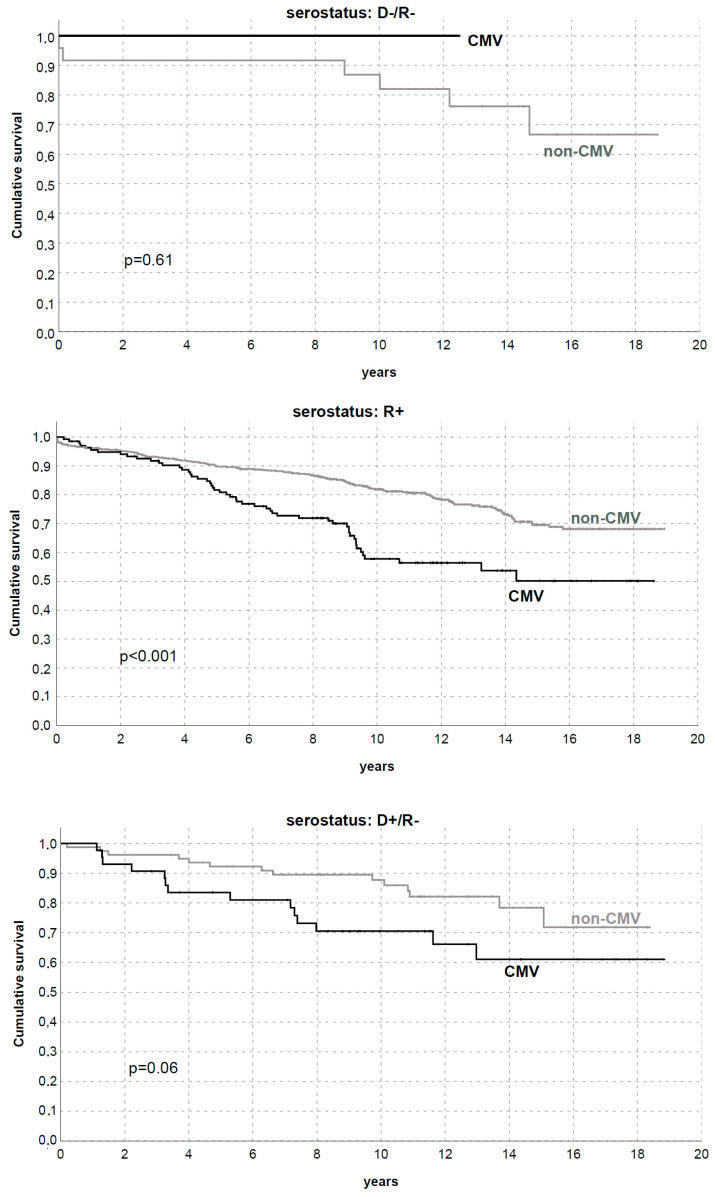
Death-censored graft survival by CMV replication/disease in the first year after transplantation with different donor and recipient CMV serostatus.

**Figure 4:**
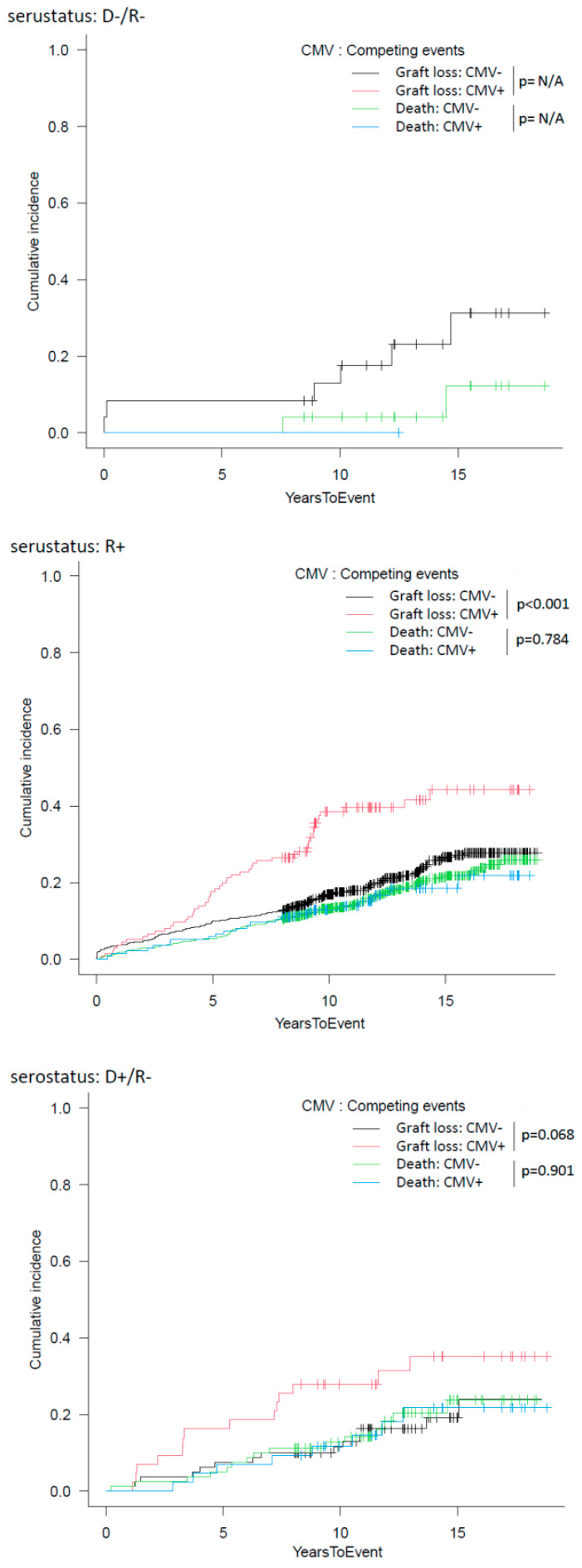
Cumulative incidence of graft loss, accounting for death as a competing risk, in patients with/without CMV replication/disease in the first year after transplantation stratified for donor and recipient CMV serostatus.

Subsequently, we divided the studied population according to CMV donor/recipient serostatus in D−/R− (Group 1, *n* = 25 [2.7%]), R+ (Group 2, *n* = 781 [84%]), and D+/R− (Group 3, *n* = 123 [13.3%]). CMV influenced graft survival only in Group 2, probably due to the low sample sizeand the low number of events in the other subgroups (Figure 3 and Figure 4).

The influence of CMV on graft survival was highlighted in Group 2, where 5- and 10-year DCGS was 89.6/80.9% and 81.6/57.7% in patients without/with CMV occurrence in the first year, respectively (*p* < 0.01, Figure 3 and Figure 4).

Considering the number of ECDs in our population with high recipient and donor ages, we also analyzed the possible interaction between CMV occurrence and donor age. Table 3 summarizes sample sizesand main characteristics of KTs stratified for donor age (<50, 50–69, and ≥70 years).

**Figure 5:**
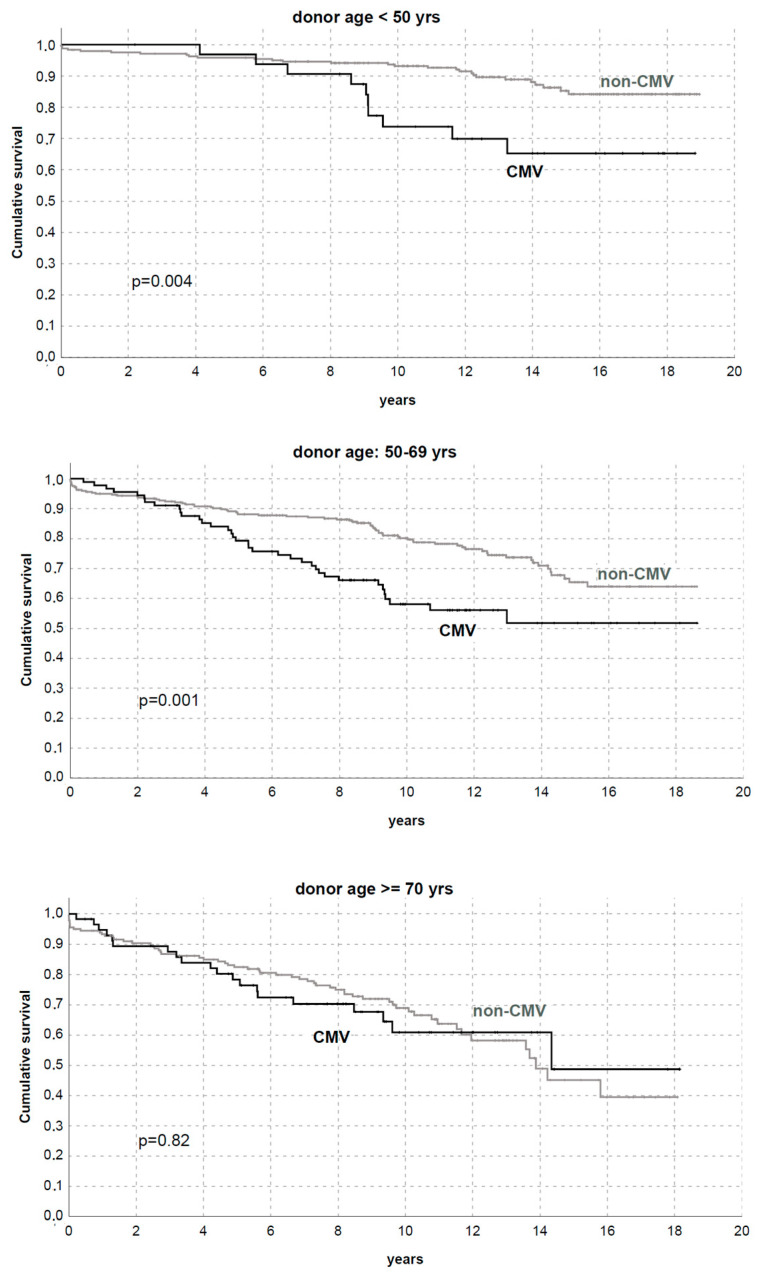
Death-censored graft survival by CMV replication/disease in the first year after transplantation with donors <50 years, 50–69 years, or ≥70 years.

**Figure 6:**
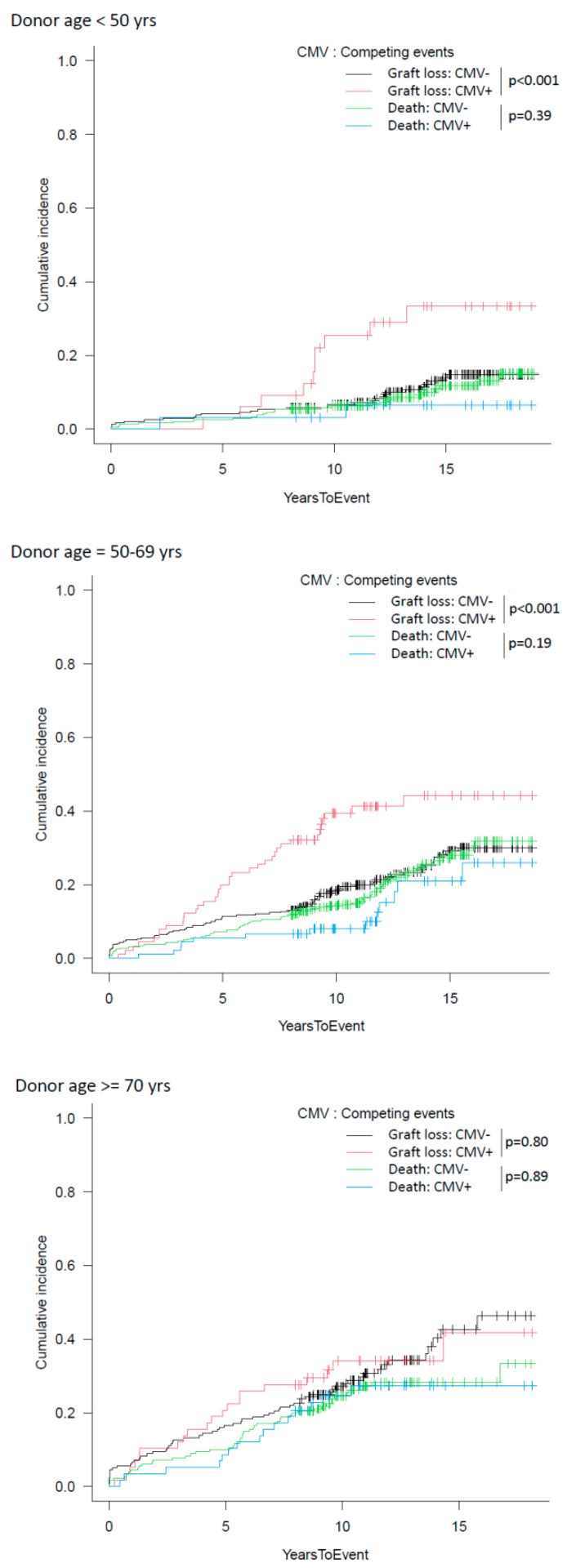
Cumulative incidence of graft loss, accounting for death as a competing risk, in patients with/without CMV replication/disease in the first year after transplantation stratified for donors <50 years, 50–69 years, or ≥70 years.

In this sub-analysis, CMV incidence significantly differed between groups (11.9% in KTs with donors < 50 years, 21.8% with donors 50–70 years, and 24.3% with donors ≥ 70 years [*p* < 0.01]), and patients with CMV history in the first year had lower graft survival in KTs with donors < 50 years and 50–69 years (*p* < 0.01), without apparent effects on recipients of grafts from donors ≥ 70 years (Figure 5 and Figure 6).

The subsequent analysis also confirmed that patients without CMV showed different DCGS by donor age (*p* < 0.01).

**Figure 7:**
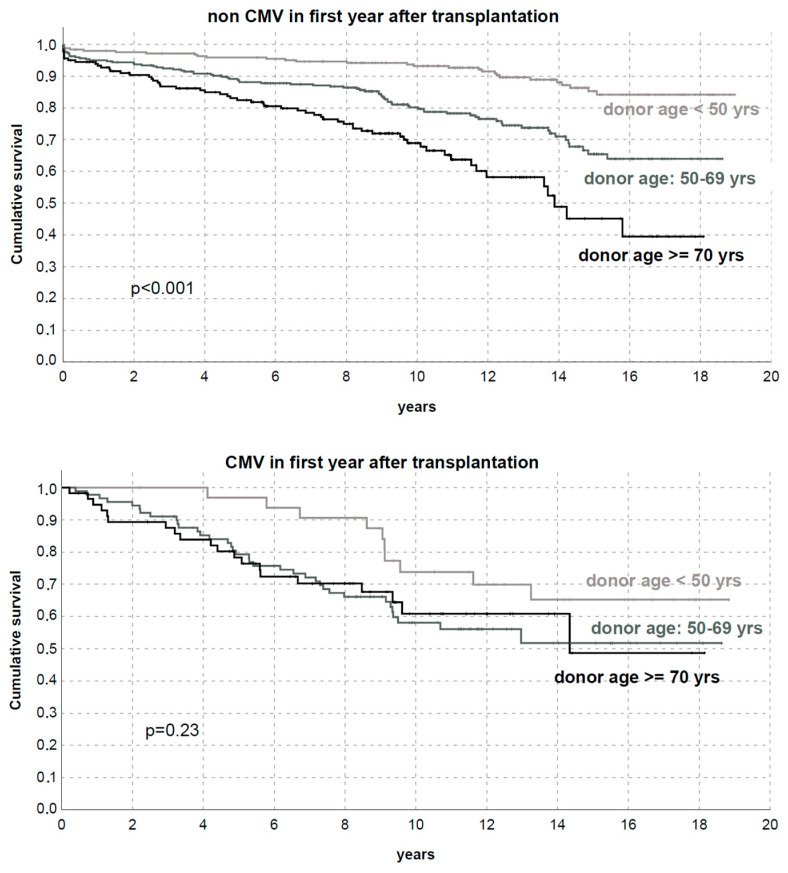
Death-censored graft survival by donor age (<50 years, 50–69 years, or ≥70 years) with or without CMV replication/disease in the first year after transplantation.

In contrast, no significant difference in DCGS by donor age was found in KTs with CMV history (*p* = 0.23, Figure 7).

In a multivariate model with the main predictors of graft survival, CMV viremia and biopsy-proven acute rejection (both in the first year after transplant), 1-year proteinuria ≥ 0.5 g/day, donor age ≥ 50 years, 1-year eGFR < 44 mL/min/1.73 m^2^/1.73 m^2^ were independently associated with death-censored graft survival (Table 4).

## 4. Discussion

Long-term graft survival and developing strategies to control and overcome modifiable risk factors are among the main challenges for transplant teams worldwide. This study focused on the CMV role in a large population of consecutive KT patients adopting pre-emptive therapy with a significant prevalence of suboptimal donors.

CMV is one of the herpesviruses that may cause significant infections in immunocompromised patients, including KTs [11,12]. In the general population, CMV infection occurs during infancy without substantial symptoms, and the virus remains in a latent state. A potentially harmful disease may develop in subjects with compromised immune defense (e.g., treatments with immunosuppressive drugs, cancers with active disease and concomitant therapy) or in women during pregnancy where CMV replication could cause fetal malformations [11,13]. In transplant patients, similar to other viruses, CMV infection occurs when a significant imbalance between the host immune system and the virus has been established, mainly during the more pronounced immunosuppressive “pressure” in the first period after transplant [11,12,14]. The direct correlation between immunosuppressive load and infection was confirmed by the incidence peak of CMV viremia/disease worldwide documented early after transplant (commonly during the first six months) but also in the period immediately after an increase in immunosuppressive treatment or severe immune impairment for different reasons (e.g., treatment of acute rejection, severe contemporary infection, an unintentional increase in drug levels for intestinal problems) [4].

Although CMV is a well-known detrimental factor for transplant outcomes, beyond the current guidelines, real-world data for the available preventive strategies and their effect on CMV occurrence are limited [6]. Also, risk factors could be debatable, with different impacts according to specific population characteristics.

Our analysis shows CMV is associated with donor and recipient age, donor pre-transplant hypertension, DR antigen compatibility, and cold ischemia time.

All these factors have been previously reported in the literature [5,15,16,17,18,19]. For example, regarding one of the main focuses of our analysis, Hemmersbach-Miller et al. documented that the risk of CMV reactivation in CMV-seropositive recipients was significantly more frequent in the older group (71.4% vs. 44.4%, *p* = 0.003) and occurred earlier (*p* = 0.003); additionally, in their multivariate model, recipient age was associated with CMV reactivation (OR, 2.48, *p* = 0.03). Old patients maintained their increased risk also when correcting for ATG induction (OR, 3.81, *p* = 0.014); interestingly, while the older group had a higher risk of infection after the initial episode, the relative hazards were approximately equal, suggesting the importance of avoiding the first episode of reactivation. The authors concluded that universal or hybrid prophylaxis should be indicated in CMV-seropositive kidney transplant recipients aged ≥65 years [15]. Our study emphasizes these associations, confirming their role in a population with a significant percentage of expanded-criteria donors and high donor/recipient age

In our analysis, CMV viremia also determined an increased risk of biopsy-proven acute rejection, new-onset diabetes (NODAT), cardiovascular complications, renal artery stenosis, and infections. All these conditions have been previously documented as related to CMV infection [14,16,20,21,22,23,24,25,26,27,28,29,30,31,32,33]. The relationship between CMV and acute rejection has been intensively studied and debated. In a multiple time-dependent Cox analysis, Sagedal et al. showed that CMV infection and CMV disease were significant independent predictors for acute clinical rejections. CMV disease, but not CMV infection, was also a predictor of tubulointerstitial rejection [31]. By contrast, Erdbruegger et al. noted that patients with CMV were more likely to receive clinically indicated biopsies, but this did not translate into a more significant number of patients with episodes of acute cellular rejection on histopathology; the additional analysis of protocol biopsies revealed a significantly higher number of episodes of rejection per patient with CMV infection, but only in a subgroup of patients with triple immunosuppression [34]. These differences may derive from the heterogeneity in donor and recipient age and criteria for acute rejection diagnosis (clinical/histological). An additional explanation could be the adoption of different strategies for kidney biopsies (clinically indicated vs. per protocol).

The association of CMV with NODAT has been extensively investigated. Hjelmesæth et al. found an incidence of new-onset diabetes of 6% in a control group of recipients without CMV infection and 26% in the group with asymptomatic CMV infection; interestingly, the group of patients with CMV infection had a significantly lower median insulin release than controls [33]. However, some groups do not confirm this association [35,36]. Once again, differences in patient characteristics, CMV prophylaxis, and immunosuppressive therapy may explain these opposite results.

Fewer studies have commented on the association between CMV and renal artery stenosis. Pouria et al. identified more CMV infections in patients with renal artery stenosis than in controls [37]. This association was confirmed by Kamali et al., who additionally noted that positive CMV-antibody was more frequent in recipients with renal artery stenosis [28].

More recently, the role of CMV in cardiovascular disease has raised attention. Courivaud et al., using a Cox regression analysis, documented that patients with post-transplantation CMV replication had an increased risk of atherosclerotic events and death [38]. Rodríguez-Goncer et al. reviewed the association of CMV with post-transplant atherosclerotic events, highlighting the ability of CMV to promote a local inflammatory milieu with dysfunction of endothelial and smooth muscle cells, accelerating immunosenescence, activating the coagulation cascade, and impairing lipid metabolism, leading to oxidative stress and cholesterol accumulation in the vessel wall. According to seroepidemiological studies in the general population, R+ patients also have an increased risk of cardiovascular disease. CMV DNA is also more commonly detected in vessel-wall specimens obtained from patients with atherosclerosis than in controls [27].

The association of CMV with other infections is well established. For example, CMV infection has been linked with an increased risk of bacterial infections (including pneumonia and nocardiosis) and invasive fungal diseases such as aspergillosis and reactivation of latent β herpesviruses such as human herpesvirus 6 and 7 [39].

Our finding that CMV could be related to urological problems is instead uncommon: to the best of our knowledge, this observation was only recently suggested by Herrera et al. [40]. In that study, the authors noted increased nephrostomy requirements in recipients with CMV, leading to their speculation that the need for nephrostomy is more common in patients with post-transplant complications with a heightened state of immunosuppression. Our population could support this association, considering that older donors and recipients are more prone to urological problems [41].

The relationship between CMV and lower eGFR has been extensively investigated. Erdbruegger et al. reported differences in renal function within the first six weeks after transplantation between patients with and without a history of CMV, with the best renal function in patients without a history of acute rejection or CMV. In contrast, patients with both acute rejection and CMV had the worst renal function [30]. In a retrospective cohort of CMV-seropositive KTs receiving ATG induction therapy, Reusing et al. showed that patients with CMV disease had more deceased donors, higher donor age, lower lymphocyte count, and lower median eGFR at day 90 [42]. Blazquez-Navarro et al. showed that CMV viral loads over 10,000 copies·mL^−1^ led to significant GFR impairment [16].

Some authors also proposed that patients with impaired graft function have a higher prevalence of CMV occurrence [30], probably due to altered pharmacokinetics/pharmacodynamics of immunosuppressive drugs [29] or impaired T-cell and immune response [43]. In this context, CMV and IF/TA, a common cause of late allograft dysfunction and proteinuria [44], have a mutual relationship, because CMV could directly determine this condition [45] but could also be more observed in patients with early IF/TA [30]. Our observation of a significant correlation between CMV and proteinuria may be included in this complex picture involving several donors, recipients, and transplant factors influencing long-term transplant function and survival.

CMV incidence and impact on the graft have also been related to D/R serostatus. Usually, patients with negative serology before kidney transplant (R−) who received a graft from a CMV-Ig-positive donor (D+) experienced severe episodes of infection with the worst outcome. With no prevention, almost all recipients with D+/R− combination develop CMV viremia (which, in these cases, indicates a primary infection), and half experience clinical symptoms. The cluster of R− patients represents a niche of high-risk subjects in which CMV infection may also occur after transfusion and sexual activity with CMV-Ig-positive partners [4,6,11,12,14].

In our analysis, we noticed that CMV occurrence had a major presence among D+/R+ and D−/R+, with a trend in D+/R–, probably due to the low sample size, despite a possible effect of the prophylaxis in limiting the severity of CMV replication/disease, as already observed by other authors [46,47].

However, in addition to these findings, our study is the first to our knowledge in a real-world setting to show a complex relationship between donor age and CMV: viral replication/disease remained an independent predictor of DCGS (HR 1.73) but differently impacted KTs from older donors. These data were also confirmed by analyzing the cumulative incidence of graft loss and considering death as a competing risk.

Despite CMV and graft loss being linked together in different case series [48,49,50,51], even adopting a multivariate model [9], and a recent metanalysis that derived a pooled OR of nearly 2-fold increased risk of kidney failure after CMV infection [7], insufficient data are available in KTs receiving ECDs, where immunological modifications may theoretically increase the risk of infection.

In our experience, CMV occurred more frequently in older donors (11.9% in KTs with donors < 50 years, 21.8% with donors 50–70 years, and 24.3% with donors ≥ 70 years) but negatively impacted graft survival only in KTs with donors < 50 years or between 50 and 69 years.

As exposed in recent guidelines about CMV management, an association between CMV and patient survival is described in many (but not all) studies, and CMV is considered a long-term allograft failure [52]. In the study by Arthurs et al., D+/R− who developed tissue-invasive CMV disease after antiviral prophylaxis showed increased allograft loss or mortality but in a population with prevalent living donors and low recipient age (47 ± 13 years) [9]. In the randomized clinical trial by Kliem et al., universal prophylaxis significantly increased long-term graft survival four years post-transplant, with the lowest graft loss rate in D+/R+ following prophylaxis. Once again, the population predominantly consisted of young donors and recipients (48.8 ± 16.1 years and 48.6 ± 14.8 years in the prophylaxis and pre-emptive group, respectively) [53]. Also, in more recent experiments correlating CMV to graft loss in different settings, donor and recipient ages reflect low utilization of old donors [8,42,54,55]. For example, in Bestard et al., the recipient age in patients with CMV disease was 38.8 ± 13 vs. 48.5 ± 13 in patients without the disease [54]. In Kaminski et al., the mean age was 48.3 ± 14.1 years in patients with late-onset disease, 47.4 ± 15.1 in the early-onset group, and 45.5 ± 15.7 in patients without CMV infection; the number of ECDs was 12/24, 14/27, and 19/71, respectively [55].

The discrepancy between increased incidence and reduced impact on allograft function requires further analysis and confirmation. However, recipients with younger donors have different and prominent immunological activation after CMV infection. Some research in the immunological area suggests different relationships between CMV, age, and immunological system. Campos et al., analyzing the effect of CMV seropositivity and aging on the distribution of NK cell subsets, identified that CMV seropositivity in young individuals does not significantly affect peripheral blood NK cell percentages and NK cell subsets defined by the use of CD56 and CD16 markers, but, in contrast, a significant increase in the rate of NK cells is observed in elderly donors, all of whom are CMV ser-positive when compared with young CMV-seropositive subjects [56]. T cell receptor clonal diversity of memory cells may become skewed in older individuals with massive expansions of cells specific for chronic infections, including CMV [57]. Based on this immunological viewpoint [55,56,57], CMV may emerge as a preeminent risk factor in this niche.

Even though our study has some limitations (lack of quantitative data on CMV viral load and timing of CMV infection during the first year to understand the cause–effect relationship with other complications), it analyzed a homogeneous population of consecutive first single KTs with an extended follow-up performed by the same transplant team (surgeons, nephrologists, and pathologists), including a significant proportion of KTs from ECDs, commonly excluded from other available experiments.

Although it is universally accepted that high-risk patients (D+/R−) should undergo extensive anti-CMV prophylaxis after kidney transplantation, uncertainties remain on managing medium-risk patients that now represent the majority of KT recipients. In particular, the beneficial effects of prophylaxis over pre-emptive therapy or a deferred approach in these patients may be weighted based on increased toxicity, especially leukopenia, given prolonged antiviral exposure (with the consequent need to reduce some class of drugs and risk of under-immunosuppression). Moreover, even if prophylaxis efficiently blocks CMV replication, it may interfere with developing a CMV-specific immune response, potentially resulting in CMV disease after cessation [58,59].

## 5. Conclusions

Based on our work, certain subgroups of patients with young donor age and specific risk factors (i.e., early signs of graft dysfunction, including mild proteinuria) should receive more extensive CMV screening or be evaluated for anti-CMV prophylaxis. These targeted interventions may reduce the CMV burden in KT recipients and improve long-term allograft function.

## Figures and Tables

**Table 1:** Main characteristics of patients with or without CMV replication/disease in the first year after transplantation.

	CMV within the First Year	NO CMV within the First Year	*p*-Value
Donor characteristics			
Hypertension, %	57.2	46.5	0.01
Diabetes mellitus, %	10.2	7.8	0.33
Donor age ≥ 60 yrs, %	62.4	46.4	<0.01
Median donor age (25th–75th percentiles)	66 yrs (53.5–72)	58 yrs (46–69)	<0.01
Median donor eGFR CKD-EPI mL/min/1.73 m^2^/1.73 m^2^ (interquartile range)	92.44 (68.89–104.35)	93.00 (72.35–110.82)	0.21
Recipient characteristics			
M/F ratio, %	66.9/33.1	64.3/35.7	0.55
Median age (25th–75thpercentiles)	58 yrs (49; 65)	54 yrs (46; 63)	0.01
HD/PD, %	70.6/29.4	78.9/21.1	0.02
Hypertension, %	91	87	0.16
Pre-Tx cardiopathy, %	40.0	33.2	0.10
Pre-Tx HCV+, %	2.3	6.1	0.06
Pre-Tx diabetes mellitus, %	14.4	11.1	0.25
Transplant characteristics			
DR 0/1/2 (match), %	0/64.6/35.4	0/54/45.9	0.03
Cold ischemia ≥ 18 h, %	57.2	49.4	0.04
Delayed graft function, %	28.7	24.7	0.29
ATG induction therapy, %	1.2	0.9	0.31

**Table 2:** Post-transplant complications, renal function, and proteinuria in patients with or without CMV replication/disease in the first year after transplantation.

	CMV within the First Year	NO CMV within the First Year	*p*-Value
1-year proteinuria ≥ 0.5 g/day, %, %	22.3	14.2	0.02
1-year eGFR (CKD-EPI) < 45 mL/min/1.73 m^2^/1.73 m^2^, %	65.3	46.5	<0.01
BPAR in 1st year, %	10.5	4.4	<0.01
NODAT in 1st year, %	27.2	18.9	0.02
De novo/recurrent glomerulonephritis, %	6.1	8.7	0.29
Urologic complication in 1st year, %	24.3	13.5	<0.01
Renal artery stenosis in 1st year, %	15.5	6.3	<0.01
Major cardiovascular complication in 1st year, %	27.6	15.1	<0.01
Ischemic cardiopathy in 1st year, %	1.7	1.6	0.95
Infection in 1st year, %	53	32.2	<0.01

BPAR: Biopsy-proven acute rejection; NODAT: New-onset diabetes mellitus after transplant.

**Table 3:** Main characteristics of patients with different donor ages.

All Patients (929 pts)	Donors < 50 yrs (277 pts)	Donors 50–69 yrs (413 pts)	Donors ≥ 70 yrs (239 pts)	*p*-Value
Donor characteristics				
eGFR CKD-EPI mL/min/1.73 m^2^/1.73 m^2^ (25–75 percentiles)	112.45 (80.2–127.25)	94.27 (69.15–104.69)	87.64 (64.41–94.28)	<0.01
Hypertension, %	20.6	56	67.6	<0.01
Diabetes mellitus, %	3.6	12.1	7.3	<0.01
Recipient characteristics				
M/F ratio, %	64.6/35.4	62.5/37.5	69/31	0.24
HD/PD, %	75/25	71/29	66/34	0.05
Pre-Tx diabetes mellitus, %	6	11.5	18.7	<0.01
Pre-Tx hypertension, %	84.7	88.7	89.9	0.16
Pre-Tx cardiopathy, %	27.7	35.5	40.9	<0.01
Pre-Tx HCV+, %	4.5	5.6	6	0.73
Transplant characteristics				
DR 0/1/2 (match), %	0/49/51	0/56/44	0/65/35	<0.01
Cold ischemia ≥ 18 h, %	37.6	55.2	58.8	<0.01
Delayed graft function, %	20.2	25.3	32.2	0.01
ATG induction therapy, %	2.4	1.3	0.5	0.18
Transplant complications				
1-year proteinuria ≥ 0.5 g/day, %	10.2	17.3	20.4	<0.01
1-year eGFR (CKD-EPI) < 45 mL/min/1.73 m^2^/1.73 m^2^, %	18.0	58.9	73.7	<0.01
BPAR in 1st year, %	6.1	5.8	4.6	0.73
NODAT in 1st year, %	13.4	22.5	21.8	<0.01
De novo/recurrent glomerulonephritis, %	10.8	6.3	8.4	0.1
Urologic complications in 1st year, %	9	19.1	17.2	<0.01
Renal artery stenosis in 1st year, %	1.1	9.4	13.8	<0.01
Major cardiovascular complications in 1st year, %	20.2	15.6	13.4	0.32
Ischemic cardiopathy in 1st year, %	0.7	1.7	2.5	0.27
Infections in 1st year, %	32.1	35.8	41.8	0.07

BPAR: Biopsy-proven acute rejection; NODAT: New-onset diabetes mellitus after transplant.

**Table 4:** Multivariate analysis for determinants of graft outcome (death-censored).

	*p*-Value	Hazard Ratio (Confidence Interval 95%)
1-year proteinuria 0.2–0.5 g/day	0.32	1.17 (0.86–1.60)
1-year proteinuria ≥ 0.5 g/day	<0.01	2.74 (1.97–3.81)
1-year eGFR (CKD-EPI) ≥ 44 mL/min/1.73 m^2^/1.73 m^2^	<0.01	0.40 (0.29–0.55)
Donor age 50–69 years	<0.01	1.66 (1.14–2.43)
Donor age ≥ 70 years	<0.01	2.13 (1.40–3.23)
CMV replication/disease in 1st year	<0.01	1.73 (1.30–2.30)
CMV IgG+ pre-transplantation (R+)	0.44	0.87 (0.61–1.44)
BPAR in 1st year	0.03	1.59 (1.05–2.39)

BPAR: Biopsy-proven acute rejection

## Data Availability

The data supporting this study’s findings are available from the corresponding author upon reasonable request.

## References

[1] Cippà P.E. (2019). New ideas for old problems: How scientific advances can change the future of organ transplantation. Transpl. Int..

[2] Freeman R.B. (2009). The “Indirect” effects of cytomegalovirus infection: Minireview. Am. J. Transplant..

[3] Reischig T., Kacer M., Hruba P., Jindra P., Hes O., Lysak D., Bouda M., Viklicky O. (2017). The impact of viral load and time to onset of cytomegalovirus replication on long-term graft survival after kidney transplantation. Antivir. Ther..

[4] Mella A., Mariano F., Dolla C., Gallo E., Manzione A.M., Di Vico M.C., Cavallo R., De Rosa F.G., Costa C., Biancone L. (2022). Bacterial and Viral Infection and Sepsis in Kidney Transplanted Patients. Biomedicines.

[5] Helanterä I., Schachtner T., Hinrichs C., Salmela K., Kyllönen L., Koskinen P., Lautenschlager I., Reinke P. (2014). Current characteristics and outcome of cytomegalovirus infections after kidney transplantation. Transpl. Infect. Dis..

[6] Kotton C.N., Kumar D., Caliendo A.M., Huprikar S., Chou S., Danziger-Isakov L., Humar A. (2018). The Third International Consensus Guidelines on the Management of Cytomegalovirus in Solid-organ Transplantation. Transplantation.

[7] Raval A.D., Kistler K.D., Tang Y., Murata Y., Snydman D.R. (2021). Epidemiology, risk factors, and outcomes associated with cytomegalovirus in adult kidney transplant recipients: A systematic literature review of real-world evidence. Transpl. Infect. Dis..

[8] Leeaphorn N., Garg N., Thamcharoen N., Khankin E.V., Cardarelli F., Pavlakis M. (2019). Cytomegalovirus mismatch still negatively affects patient and graft survival in the era of routine prophylactic and preemptive therapy: A paired kidney analysis. Am. J. Transplant..

[9] Arthurs S.K., Eid A.J., Pedersen R.A., Kremers W.K., Cosio F.G., Patel R., Razonable R.R. (2008). Delayed-onset primary cytomegalovirus disease and the risk of allograft failure and mortality after kidney transplantation. Clin. Infect. Dis..

[10] Schuster N.A., Hoogendijk E.O., Kok A.A.L., Twisk J.W.R., Heymans M.W. (2020). Ignoring competing events in the analysis of survival data may lead to biased results: A nonmathematical illustration of competing risk analysis. J. Clin. Epidemiol..

[11] Fishman J.A. (2017). Infection in Organ Transplantation. Am. J. Transplant..

[12] Haidar G., Boeckh M., Singh N. (2021). Cytomegalovirus infection in solid organ and hematopoietic cell transplantation: State of the evidence. J. Infect. Dis..

[13] Davis N.L., King C.C., Kourtis A.P. (2017). Cytomegalovirus infection in pregnancy. Birth Defects Res..

[14] Kaminski H., Fishman J.A. (2016). The Cell Biology of Cytomegalovirus: Implications for Transplantation. Am. J. Transplant..

[15] Hemmersbach-Miller M., Alexander B.D., Pieper C.F., Schmader K.E. (2020). Age matters: Older age as a risk factor for CMV reactivation in the CMV serostatus–positive kidney transplant recipient. Eur. J. Clin. Microbiol. Infect. Dis..

[16] Blazquez-Navarro A., Dang-Heine C., Wittenbrink N., Bauer C., Wolk K., Sabat R., Westhoff T.H., Sawitzki B., Reinke P., Thomusch O. (2018). BKV, CMV, and EBV Interactions and their Effect on Graft Function One Year Post-Renal Transplantation: Results from a Large Multi-Centre Study. EBioMedicine.

[17] Schlott F., Steubl D., Hoffmann D., Matevossian E., Lutz J., Heemann U., Hösel V., Busch D.H., Renders L., Neuenhahn M. (2017). Primary cytomegalovirus infection in seronegative kidney transplant patients is associated with protracted cold ischemic time of seropositive donor organs. PLoS ONE.

[18] Kirisri S., Vongsakulyanon A., Kantachuvesiri S., Razonable R.R., Bruminhent J. (2021). Predictors of CMV Infection in CMV-Seropositive Kidney Transplant Recipients: Impact of Pretransplant CMV-Specific Humoral Immunity. Open Forum Infect. Dis..

[19] Fernández-Ruiz M., Corrales I., Amat P., González E., Andrés A., Navarro D., Aguado J.M. (2015). Influence of Age and HLA Alleles on the CMV-Specific Cell-Mediated Immunity among CMV-Seropositive Kidney Transplant Candidates. Am. J. Transplant..

[20] Bischof N., Wehmeier C., Dickenmann M., Hirt-Minkowski P., Amico P., Steiger J., Naegele K., Hirsch H.H., Schaub S. (2020). Revisiting cytomegalovirus serostatus and replication as risk factors for inferior long-term outcomes in the current era of renal transplantation. Nephrol. Dial. Transplant..

[21] Jorgenson M.R., Descourouez J.L., Cardinale B., Lyu B., Astor B.C., Garg N., Saddler C.M., Smith J.A., Mandelbrot D.A. (2019). Risk of opportunistic infection in kidney transplant recipients with cytomegalovirus infection and associated outcomes. Transpl. Infect. Dis..

[22] Samson L.D., van den Berg S.P.H., Engelfriet P., Boots A.M.H., Hendriks M., de Rond L.G.H., de Zeeuw-Brouwer M., Verschuren W.M., Borghans J.A.M., Buisman A.M. (2020). Limited effect of duration of CMV infection on adaptive immunity and frailty: Insights from a 27-year-long longitudinal study. Clin. Transl. Immunol..

[23] Hjelmesæth J., Midtvedt K., Jenssen T., Hartmann A. (2001). Insulin Resistance After Renal Transplantation. Diabetes Care.

[24] Einollahi B., Motalebi M., Salesi M., Ebrahimi M., Taghipour M. (2014). The impact of cytomegalovirus infection on new-onset diabetes mellitus after kidney transplantation: A review on current findings. J. Nephropathol..

[25] Alnsasra H., Asleh R., Kumar N., Lopez C., Toya T., Kremers W.K., Edwards B., Daly R.C., Kushwaha S.S. (2021). Incidence, Risk Factors, and Outcomes of Stroke Following Cardiac Transplantation. Stroke.

[26] Van Laecke S., Malfait T., Schepers E., Van Biesen W. (2018). Cardiovascular disease after transplantation: An emerging role of the immune system. Transpl. Int..

[27] Rodríguez-Goncer I., Fernández-Ruiz M., Aguado J.M. (2020). A critical review of the relationship between post-transplant atherosclerotic events and cytomegalovirus exposure in kidney transplant recipients. Expert Rev. Anti. Infect. Ther..

[28] Kamali K., Abbasi M.A., Behzadi A.H., Mortazavi A., Bastani B. (2010). Incidence and risk factors of transplant renal artery stenosis in living unrelated donor renal transplantation. J. Ren. Care.

[29] Reischig T., Kacer M., Hes O., Machova J., Nemcova J., Lysak D., Jindra P., Pivovarcikova K., Kormunda S., Bouda M. (2019). Cytomegalovirus prevention strategies and the risk of BK polyomavirus viremia and nephropathy. Am. J. Transplant..

[30] Erdbrügger U., Scheffner I., Mengel M., Schwarz A., Haller H., Gwinner W. (2015). Long-term impact of CMV infection on allografts and on patient survival in renal transplant patients with protocol biopsies. Am. J. Physiol.-Ren. Physiol..

[31] Sagedal S., Nordal K.P., Hartmann A., Sund S., Scott H., Degré M., Foss A., Leivestad T., Osnes K., Fauchald P. (2002). The impact of cytomegalovirus infection and disease on rejection episodes in renal allograft recipients. Am. J. Transplant..

[32] Toupance O., Bouedjoro-Camus M.C., Carquin J., Novella J.L., Lavaud S., Wynckel A., Jolly D., Chanard J. (2000). Cytomegalovirus-related disease and risk of acute rejection in renal transplant recipients: A cohort study with case-control analyses. Transpl. Int..

[33] Hjelmesæth J., Sagedal S., Hartmann A., Rollag H., Egeland T., Hagen M., Nordal K.P., Jenssen T. (2004). Asymptomatic cytomegalovirus infection is associated with increased risk of new-onset diabetes mellitus and impaired insulin release after renal transplantation. Diabetologia.

[34] Erdbruegger U., Scheffner I., Mengel M., Schwarz A., Verhagen W., Haller H., Gwinner W. (2012). Impact of CMV infection on acute rejection and long-term renal allograft function: A systematic analysis in patients with protocol biopsies and indicated biopsies. Nephrol. Dial. Transplant..

[35] Abou-Ayache R., Bchler M., Le Pogamp P., Westeel P.F., Le Meur Y., Etienne I., Hurault De Ligny B., Toupance O., Caillard S., Sinnasse-Raymond G. (2011). The influence of cytomegalovirus infections on patient and renal graft outcome: A 3-year, multicenter, observational study (post-ECTAZ study). Transplant. Proc..

[36] Dedinská I., Laca L., Miklušica J., Kantárová D., Galajda P., Mokáň M. (2016). Correlation between CMV infection and posttransplantation new-onset diabetes mellitus. Int. J. Organ Transplant. Med..

[37] Pouria S., State O.I., Wong W., Hendry B.M. (1998). CMV infection is associated with transplant renal artery stenosis. QJM-Mon. J. Assoc. Physicians.

[38] Courivaud C., Bamoulid J., Chalopin J.M., Gaiffe E., Tiberghien P., Saas P., Ducloux D. (2013). Cytomegalovirus exposure and cardiovascular disease in kidney transplant recipients. J. Infect. Dis..

[39] Roman A., Manito N., Campistol J.M., Cuervas-Mons V., Almenar L., Arias M., Casafont F., del Castillo D., Crespo-Leiro M.G., Delgado J.F. (2014). The impact of the prevention strategies on the indirect effects of CMV infection in solid organ transplant recipients. Transplant. Rev..

[40] Herrera S., Bernal-Maurandi J., Cofan F., Ventura P., Marcos M.A., Linares L., Cuesta G., Diekmann F., Moreno A., Bodro M. (2021). BK virus and cytomegalovirus coinfections in kidney transplantation and their impact on allograft loss. J. Clin. Med..

[41] Bruintjes M.H.D., D’ancona F.C.H., Zhu X., Hoitsma A.J., Warlé M.C. (2019). An update on early urological complications in kidney transplantation: A national cohort study. Ann. Transplant..

[42] Reusing J.O., Feitosa E.B., Agena F., Pierrotti L.C., Azevedo L.S.F., Kotton C.N., David-Neto E. (2018). Cytomegalovirus prophylaxis in seropositive renal transplant recipients receiving thymoglobulin induction therapy: Outcome and risk factors for late CMV disease. Transpl. Infect. Dis..

[43] Jamal A.J., Husain S., Li Y., Famure O., Kim S.J. (2014). Risk factors for late-onset cytomegalovirus infection or disease in kidney transplant recipients. Transplantation.

[44] Naesens M., Lerut E., Emonds M.P., Herelixka A., Evenepoel P., Claes K., Bammens B., Sprangers B., Meijers B., Jochmans I. (2016). Proteinuria as a Noninvasive Marker for Renal Allograft Histology and Failure: An Observational Cohort Study. J. Am. Soc. Nephrol..

[45] Reischig T., Jindra P., Hes O., Bouda M., Kormunda S., Třeška V. (2009). Effect of cytomegalovirus viremia on subclinical rejection or interstitial fibrosis and tubular atrophy in protocol biopsy at 3 months in renal allograft recipients managed by preemptive therapy or antiviral prophylaxis. Transplantation.

[46] Manuel O., Kralidis G., Mueller N.J., Hirsch H.H., Garzoni C., Van Delden C., Berger C., Boggian K., Cusini A., Koller M.T. (2013). Impact of antiviral preventive strategies on the incidence and outcomes of cytomegalovirus disease in solid organ transplant recipients. Am. J. Transplant..

[47] Stern M., Hirsch H., Cusini A., Van Delden C., Manuel O., Meylan P., Boggian K., Mueller N.J., Dickenmann M. (2014). Cytomegalovirus serology and replication remain associated with solid organ graft rejection and graft loss in the era of prophylactic treatment. Transplantation.

[48] López-Oliva M.O., Flores J., Madero R., Escuin F., Santana M.J., Bellón T., Selgas R., Jiménez C. (2017). Cytomegalovirus infection after kidney transplantation and long-term graft loss. Nefrologia.

[49] Bal Z., Uyar M.E., Tutal E., Erdogan E., Colak T., Sezer S., Haberal M. (2013). Cytomegalovirus infection in renal transplant recipients: One center’s experience. Transplant. Proc..

[50] Kanter J., Pallardó L., Gavela E., Escudero V., Beltrán S., Morales A., Ávila A., Crespo J.F. (2009). Cytomegalovirus Infection Renal Transplant Recipients: Risk Factors and Outcome. Transplant. Proc..

[51] Siodlak M., Jorgenson M.R., Descourouez J.L., Leverson G.E., Mandelbrot D.A., Smith J.A., Redfield R.R. (2018). Impact of High-Dose Acyclovir Cytomegalovirus Prophylaxis Failure in Abdominal Solid Organ Transplant Recipients. Pharmacotherapy.

[52] Razonable R.R., Humar A. (2019). Cytomegalovirus in solid organ transplant recipients—Guidelines of the American Society of Transplantation Infectious Diseases Community of Practice. Clin. Transplant..

[53] Kliem V., Fricke L., Wollbrink T., Burg M., Radermacher J., Rohde F. (2008). Improvement in long-term renal graft survival due to CMV prophylaxis with oral ganciclovir: Results of a randomized clinical trial. Am. J. Transplant..

[54] Bestard O., Lucia M., Crespo E., Van Liempt B., Palacio D., Melilli E., Torras J., Llaudõ I., Cerezo G., Taco O. (2013). Pretransplant immediately early-1-specific T cell responses provide protection for CMV infection after kidney transplantation. Am. J. Transplant..

[55] Kaminski H., Couzi L., Garrigue I., Moreau J.F., Déchanet-Merville J., Merville P. (2016). Easier Control of Late-Onset Cytomegalovirus Disease Following Universal Prophylaxis Through an Early Antiviral Immune Response in Donor-Positive, Recipient-Negative Kidney Transplants. Am. J. Transplant..

[56] Campos C., Pera A., Sanchez-Correa B., Alonso C., Lopez-Fernandez I., Morgado S., Tarazona R., Solana R. (2014). Effect of age and CMV on NK cell subpopulations. Exp. Gerontol..

[57] Vescovini R., Biasini C., Fagnoni F.F., Telera A.R., Zanlari L., Pedrazzoni M., Bucci L., Monti D., Medici M.C., Chezzi C. (2007). Massive Load of Functional Effector CD4 + and CD8 + T Cells against Cytomegalovirus in Very Old Subjects. J. Immunol..

[58] Rennie T.J.W., Geddes C.G., McIntyre-McClure R., Chua B.H.E., Metcalfe W., Johannessen I., Phelan P.J., Padmanabhan N., Clancy M.J., Black H. (2021). Efficacy and side effect profile of two CMV prophylaxis strategies in high and intermediate risk kidney transplant recipients–a multicentre national study. J. Nephrol..

[59] Hellemans R., Abramowicz D. (2022). Cytomegalovirus after kidney transplantation in 2020: Moving towards personalized prevention. Nephrol. Dial. Transplant..

